# Memristive and CMOS Devices for Neuromorphic Computing

**DOI:** 10.3390/ma13010166

**Published:** 2020-01-01

**Authors:** Valerio Milo, Gerardo Malavena, Christian Monzio Compagnoni, Daniele Ielmini

**Affiliations:** Dipartimento di Elettronica, Informazione e Bioingegneria, Politecnico di Milano and Italian Universities Nanoelectronics Team (IU.NET), Piazza L. da Vinci 32, 20133 Milano, Italy; valerio.milo@polimi.it (V.M.); gerardo.malavena@polimi.it (G.M.); christian.monzio@polimi.it (C.M.C.)

**Keywords:** neuromorphic computing, Flash memories, memristive devices, resistive switching, synaptic plasticity, artificial neural network, spiking neural network, pattern recognition

## Abstract

Neuromorphic computing has emerged as one of the most promising paradigms to overcome the limitations of von Neumann architecture of conventional digital processors. The aim of neuromorphic computing is to faithfully reproduce the computing processes in the human brain, thus paralleling its outstanding energy efficiency and compactness. Toward this goal, however, some major challenges have to be faced. Since the brain processes information by high-density neural networks with ultra-low power consumption, novel device concepts combining high scalability, low-power operation, and advanced computing functionality must be developed. This work provides an overview of the most promising device concepts in neuromorphic computing including complementary metal-oxide semiconductor (CMOS) and memristive technologies. First, the physics and operation of CMOS-based floating-gate memory devices in artificial neural networks will be addressed. Then, several memristive concepts will be reviewed and discussed for applications in deep neural network and spiking neural network architectures. Finally, the main technology challenges and perspectives of neuromorphic computing will be discussed.

## 1. Introduction

The complementary metal-oxide semiconductor (CMOS) technology has sustained tremendous progress in communication and information processing since the 1960s. Thanks to the continuous miniaturization of the metal-oxide semiconductor (MOS) transistor according to the Moore’s law [1] and Dennard scaling rules [2], the clock frequency and integration density on the chip have seen an exponential increase. In the last 15 years, however, the Moore’s scaling law has been slowed down by two fundamental issues, namely the excessive subthreshold leakage currents and the increasing heat generated within the chip [3,4]. To overcome these barriers, new advances have been introduced, including the adoption of high-k materials as the gate dielectric [5], the redesign of the transistor with multigate structures [6,7], and 3D integration [8]. Besides the difficult scaling, another crucial issue of today’s digital computers is the physical distinction between the central processing unit (CPU) and the memory unit at the origin of extensive data movement during computation, especially for data-intensive tasks [9]. Solving the memory bottleneck requires a paradigm shift in architecture, where computation is executed in situ within the data by exploiting, e.g., the ability of memory arrays to implement matrix-vector multiplication (MVM) [10,11]. This novel architectural approach is referred to as in-memory computing, which provides the basis for several outstanding applications, such as pattern classification [12,13], analogue image processing [14], and the solution of linear systems [15,16] and of linear regression problems [17].

In this context, neuromorphic computing has been receiving increasing interest for its ability to mimic the human brain. A neuromorphic circuit consists of a network of artificial neurons and synapses capable of processing sensory information with massive parallelism and ultra-low power dissipation [18]. The realization of scalable, high density, and high-performance neuromorphic circuits generally requires the extensive adoption of memory devices serving the role of synaptic links and/or neuron elements. The device structure and operation of these memory devices may require specific optimization for neuromorphic circuits.

This work reviews the current status of neuromorphic devices, with a focus on both CMOS and memristive devices for implementation of artificial synapses and neurons in both deep neural networks (DNNs) and spiking neural networks (SNNs). The paper is organized as follows: Section 2 provides an overview of the major neuromorphic computing concepts from a historical perspective. Section 3 is an overview of the operating principles of mainstream NAND and NOR Flash technologies, and their adoption in neuromorphic networks. Section 4 describes the most important memristive concepts being considered for neuromorphic computing applications. Section 5 addresses the adoption of memristive devices in DNNs and SNNs for hardware demonstration of cognitive functions, such as pattern recognition and image/face classification. Finally, Section 6 discusses issues and future perspectives for large-scale hardware implementation of neuromorphic systems with CMOS/memristive devices.

## 2. Neuromorphic Computing Concepts

**Figure 1:**
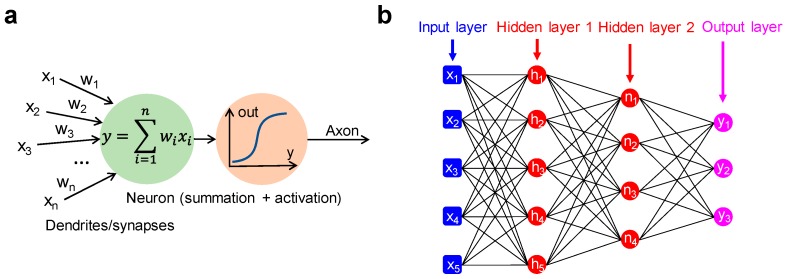
(**a**) Conceptual illustration of McCulloch and Pitts artificial neuron architecture, where the weighted sum of the input signals is subject to the application of a non-linear activation function yielding the output signal. (**b**) Schematic representation of a multilayer perceptron consisting of two hidden layers between the input and the output layer.

The origin of neuromorphic computing can be traced back to 1949, when McCulloch and Pitts proposed a mathematical model of the biological neuron. This is depicted in Figure 1a, where the neuron is conceived as a processing unit, operating (i) a summation of input signals (x_1_, x_2_, x_3_, …), each multiplied by a suitable synaptic weight (w_1_, w_2_, w_3_, …) and (ii) a non-linear transformation according to an activation function, e.g., a sigmoidal function [19]. A second landmark came in 1957, when Rosenblatt developed the model of a fundamental neural network called multiple-layer perceptron (MLP) [20], which is schematically illustrated in Figure 1b. The MLP consists of an input layer, one or more intermediate layers called hidden layers, and an output layer, through which the input signal is forward propagated toward the output. The MLP model constitutes the backbone for the emerging concept of DNNs. DNNs have recently shown excellent performance in tasks, such as pattern classification and speech recognition, via extensive supervised training techniques, such as the backpropagation rule [21,22,23]. DNNs are usually implemented in hardware with von Neumann platforms, such as the graphics processing unit (GPU) [24] and the tensor processing unit (TPU) [25], used to execute both training and inference. These hardware implementations, however, reveal all the typical limitations of the von Neumann architecture, chiefly the large energy consumption in contrast with the human brain model.

To significantly improve the energy efficiency of DNNs, MVM in crossbar memory arrays has emerged as a promising approach [26,27]. Memory devices also enable the implementation of learning schemes able to replicate the biological synaptic plasticity at the device level. CMOS memories, such as the static random access memory (SRAM) [28,29] and the Flash memory [30], were initially adopted to capture synaptic behaviors in hardware. In the last 10 years, novel material-based memory devices, generically referred to as memristors [31], have evidenced attractive features for the implementation of neuromorphic hardware, including non-volatile storage, low power operation, nanoscale size, and analog resistance tunability. In particular, memristive technologies, which include resistive switching random access memory (RRAM), phase change memory (PCM), and other emergent memory concepts based on ferroelectric and ferromagnetic effects, have been shown to achieve synapse and neuron functions, enabling the demonstration of fundamental cognitive primitives as pattern recognition in neuromorphic networks [32,33,34,35].

**Figure 2:**
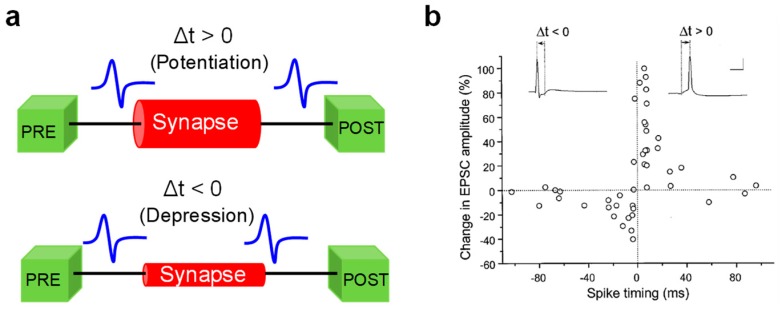
(**a**) Sketch of the spike-timing-dependent plasticity (STDP) learning rule. If the PRE spike arrives just before the POST spike at the synaptic terminal (Δt > 0), the synapse undergoes a potentiation process, resulting in a weight (conductance) increase (**top**). Otherwise, if the PRE spike arrives just after the POST spike (Δt < 0), the synapse undergoes a depression process, resulting in a weight (conductance) decrease (**bottom**). (**b**) Relative change of synaptic weight as a function of the relative time delay between PRE and POST spikes measured in hippocampal synapses by Bi and Poo. Reprinted with permission from [38]. Copyright 1998 Society for Neuroscience.

The field of neuromorphic networks includes both the DNN [36], and SNN, the latter more directly inspired by the human brain [37]. Contrary to DNNs, the learning ability in SNNs emerges via unsupervised training processes, where synapses are potentiated or depressed by bio-realistic learning rules inspired by the brain. Among these local learning rules, spike-timing-dependent plasticity (STDP) and spike-rate-dependent plasticity (SRDP) have received intense investigation for hardware implementation of brain-inspired SNNs. In STDP, which was experimentally demonstrated in hippocampal cultures by Bi and Poo in 1998 [38], the synaptic weight update depends on the relative timing between the presynaptic spike and the post-synaptic spike (Figure 2a). In particular, if the pre-synaptic neuron (PRE) spike precedes the post-synaptic neuron (POST) spike, namely the relative delay of spikes, Δt = t_post_ − t_pre_, is positive, then the interaction between the two spikes causes the synapse to increase its weight, which goes under the name of synaptic potentiation. On the other hand, if the PRE spike follows the POST spike, i.e., Δt is negative, then the synapse undergoes a weight decrease or synaptic depression (Figure 2b). In SRDP, instead, the rate of spikes emitted by externally stimulated neurons dictates the potentiation or depression of the synapse, with high and low frequency stimulation leading to synaptic potentiation and depression, respectively [39]. Unlike STDP relying on pairs of spikes, SRDP has been attributed to the complex combination of three spikes (triplet) or more [40,41,42,43]. In addition to the ability to learn in an unsupervised way and emulate biological processes, SNNs also offer a significant improvement in energy efficiency thanks to the ability to process data by transmission of short spikes, hence consuming power only when and where the spike occurs [18]. Therefore, CMOS and memristive concepts can offer great advantages in the implementation of both DNNs and SNNs, providing a wide portfolio of functionalities, such as non-volatile weight storage, high scalability, energy efficient in-memory computing via MVM, and online weight adaptation in response to external stimuli.

## 3. Mainstream Memory Technologies for Neuromorphic and Brain-Inspired Systems

### 3.1. Memory Transistors and Mainstream Flash Technologies

**Figure 3:**
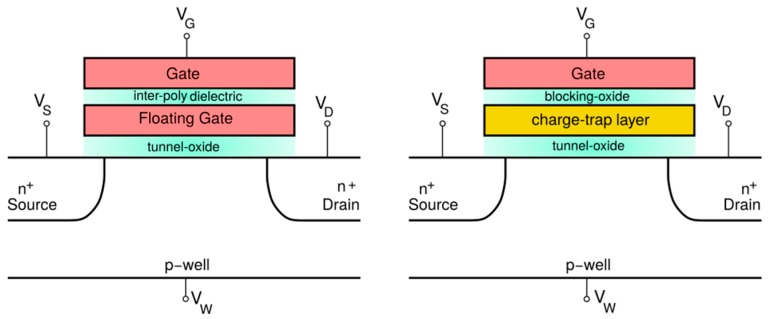
Schematic of a memory cell exploiting (**left**) a highly doped polysilicon layer and (**right**) a dielectric layer with a high density of microscopic defects for charge storage.

The memory transistor represents the elementary building unit at the basis of modern mainstream non-volatile storage technologies. It consists of a mainstream MOS transistor whose structure is modified to accommodate a charge-storage layer in its gate stack, allowing carriers to be confined in a well-defined region due to the resulting potential barriers. As shown in Figure 3, the most adopted solutions for such a layer are based either on highly doped polycrystalline silicon (polysilicon) or a dielectric material able to capture and release electrons and holes thanks to its peculiar high density of defects. The charge storage layer is usually referred to as floating gate in the former case, and charge-trap layer in the latter one. However, in both cases, storing a net charge in the memory transistor floating gate or charge-trap layer results in a shift of the drain current vs. gate voltage ($I_{DS}-V_{GS}$) curve due to the corresponding variation of the device threshold voltage ($V_{T}$). In particular, such variation is mainly ruled by the capacitance between the transistor gate and the charge-storage layer, $C_{sg}$, according to $\Delta V_{T}=-Q_{s}/C_{sg}$, meaning that a net positive or negative stored charge ($Q_{s}$) is reflected in a negative or positive $V_{T}$ shift ($\Delta V_{T}$), respectively. As a consequence, a proper discretization of the stored charge in each memory transistor allows one or multiple bits of information to be stored that can be accessed through a $V_{T}$ read operation.

**Figure 4:**
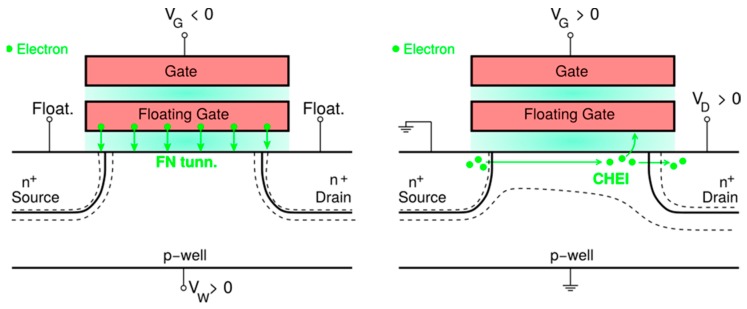
Physical mechanisms and corresponding voltage schemes exploited to change the amount of charge in the cell storage layer, consisting of (**left**) Fowler–Nordheim (FN) and (**right**) channel hot-electron injection (CHEI).

In order to reliably accomplish the tuning of the stored charge and, consequently, the modification of the information content through the program (making the stored charge more negative) and erase (making the stored charge more positive) operations, suitable physical mechanisms must be selected. As schematically depicted in Figure 4, the most widely adopted physical mechanisms are the Fowler–Nordheim (FN) tunneling, for both program and erase operations, and the channel hot electron injection (CHEI), for program operation only. In the former case, the bias voltages applied to the memory transistor contacts are chosen to generate large vertical electric fields that activate carrier exchange between the substrate and the storage layer by the quantum mechanical current through the energy barrier separating them. In the latter case, instead, CHEI is achieved by accelerating the transistor on-state current electrons by applying a positive drain-to-source voltage drop ($V_{DS}$). If $V_{DS}$ is large enough, the energy acquired by the channel electrons is sufficient for them to overcome the tunnel-oxide energy barrier and to be redirected to the charge-storage layer due to the positive $V_{GS}$. Moreover, it is worth mentioning that, for a target $\Delta V_{T}$ to be achieved over comparable time scales, CHEI requires much lower voltages to be applied with respect to FN tunneling. On the contrary, its injection efficiency is of the order of 10^−5^ only, much smaller than that of FN tunneling (very close to one). A final but important remark is that for both CHEI and FN tunneling, the maximum number of program/erase cycles that can be performed on the devices is usually smaller than 10^5^; in fact, for larger cycling doses, the number of defects generated in the tunnel oxide by the program/erase operations severely undermines the transistor reliability.

**Figure 5:**
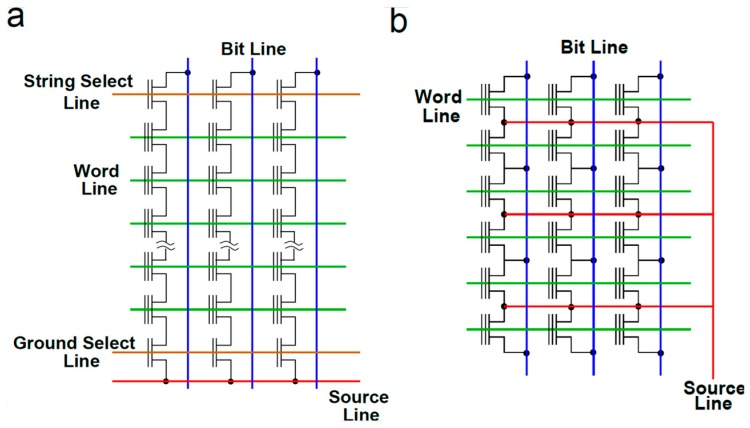
Schematic of memory arrays based on (**a**) NAND Flash and (**b**) NOR Flash architecture.

Starting from the schematic structure shown in Figure 3, the arrangement of memory transistors to build memory arrays and their working conditions are strictly related to the specific targeted application. In particular, two solutions that have ruled the non-volatile memory market since their very first introduction are the NAND Flash [44] and NOR Flash [45] architectures (Figure 5). Although they share the important peculiarity that the erase operation, exploiting FN tunneling to reduce the amount of the stored negative charge, involves a large number of cells at the same time (a block of cell), some relevant differences can be mentioned.

NAND Flash technology is the main solution for the storage of large amounts of data, therefore achieving large bit storage density, i.e., the ratio between the chip capacity and its area is a mandatory requirement. For this purpose, NAND Flash memory transistors are deeply scaled (up to a feature size as small as 15 nm) and arranged in series connection, making the memory cells belonging to each string accessible only through the contacts at their top and bottom ends (Figure 5a). In such a way, the area occupancy of each cell is minimized; on the other hand, the attempt to minimize the array fragmentation and to reduce the area occupancy of the control circuitry makes the random access time to the cells quite long (tens of μs), due to the consequent delays of the signals propagating over the long WLs and BLs. For this reason, programming schemes taking advantage of the low current and high injection efficiency of FN tunneling were developed to program many memory transistors at the same time, allowing extremely high throughputs (tens or even hundreds of Mbytes/s) to be achieved.

The NOR Flash technology, on the other hand, is mainly intended for code storage, making the storage and retrieval of small packets of data (a few bytes) as fast as possible a mandatory requirement. As a consequence, in order to make each memory cell directly accessible through dedicated contacts, the memory transistors are connected in parallel, as shown in Figure 5b. Thanks to this architecture, a fast and single-cell selective program operation can be easily achieved exploiting CHEI. From the cell design standpoint, this results in a limited channel scalability, due to the need for the cell to withstand relatively high $V_{DS}$ during its operation. Even though these features determine a larger cell footprint and, in turn, a higher cost of NOR Flash with respect to NAND Flash technologies, they allow NOR Flash arrays to guarantee a superior array reliability, being an important requirement for code storage applications.

### 3.2. Memory Transistors as Synaptic Devices in Artificial Neural Networks

The first proposal of exploiting memory transistors as artificial synapses in artificial neural networks (ANNs) and brain-inspired neural networks dates back to the 1990s directly from the pioneering work presented in ref. [46]. The basic idea proposed there is to take advantage of the subthreshold characteristic $I_{DS}-V_{GS}$ of an n-channel floating-gate memory transistor to reproduce the biologically observed synaptic behavior and to exploit it to build large-scale neuromorphic systems. In fact, when operated in a subthreshold regime, a memory transistor exhibits an $I_{DS}-V_{GS}$ relation that can be expressed as:

(1) $$I_{DS}=I_{0}\cdot exp\left[\frac{q\alpha_{G}\left(V_{GS}-V_{T}^{ref}\right)}{mkT}\right]\cdot exp\left[\frac{-q\alpha_{G}\Delta V_{T}}{mkT}\right],$$

where $I_{0}$ is the current pre-factor, *q* is the elementary charge, *m* is the subthreshold slope ideality factor, kT is the thermal energy, $\alpha_{G}$ is the gate-to-floating-gate capacitive coupling ratio, and $\Delta V_{T}$ is the floating-gate transistor $V_{T}$ shift from an arbitrary chosen $V_{T}^{ref}$.

With reference to the previous equation, $I_{DS}$ can be decomposed in the product of two contributions. The first factor, $I_{0}\cdot exp\left[\frac{q\alpha_{G}\left(V_{GS}-V_{T}^{ref}\right)}{mkT}\right]$, is a function of $V_{GS}$ only, and represents the input presynaptic signal; the remaining scaling factor, $W=exp\left[\frac{-q\alpha_{G}\Delta V_{T}}{mkT}\right]$, instead, depending on $\Delta V_{T}$ but not on $V_{GS}$, can be thought of as the synaptic weight.

**Figure 6:**
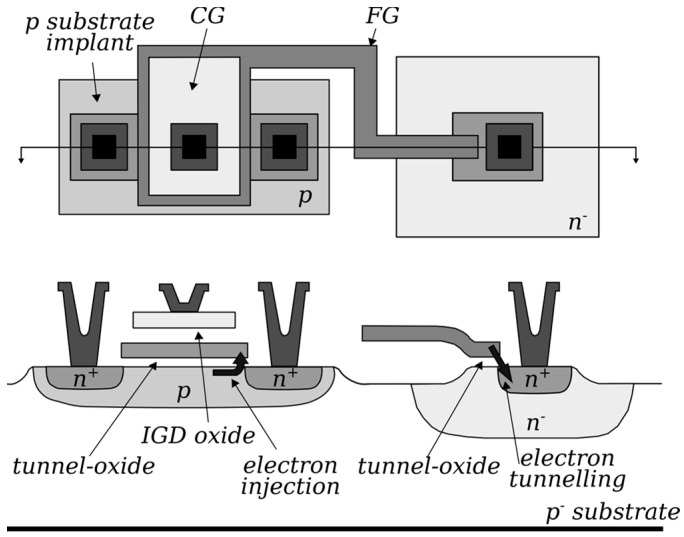
Top view (**up**) and side view (**down**) of the synaptic transistor. Physical mechanisms exploited for program (electron injection) and erase (electron tunneling) are highlighted too. Adapted with permission from [48]. Copyright 1997, IEEE.

When compared with other modern approaches based on emerging memory technologies, this solution presents the clear advantages of (i) limited power consumption, thanks to the reduced currents peculiar of transistors operated below the threshold; (ii) fine weight granularity, coming to the virtually analog and bidirectional $V_{T}$ tuning; and (iii) a mature and well-established CMOS fabrication technology. In particular, the relevance of the last point can be easily understood by considering the possibility of arranging a large number of floating-gate transistors in very dense and reliable memory arrays, normally employed for storage purposes. However, when exploited as synaptic arrays in neuromorphic applications, such memory arrays must meet the mandatory condition of single-cell selectivity during both program and erase operations, meaning that both the positive and negative tuning of the $V_{T}$ (weight) of each memory cell (synapse) must be guaranteed. Even if this consideration makes a NOR-type array inherently more suitable to be used in these fields because of its architecture that allows direct access to each cell by dedicated contacts, its standard block-erase scheme must still be overcome. For this reason, since its very first proposal, the synaptic transistor introduced in refs. [46,47,48], and tested on LTD and LTP based on the STDP learning rule in refs. [30,48], includes an additional contact with respect to standard n-channel floating-gate transistors (Figure 6) to be connected to signal lines running orthogonal to the WLs [46]. While keeping CHEI for the program, the erase operation takes place by removing stored electrons by FN tunneling when a sufficiently high electric field is developed between the tunneling contact and the transistor floating gate that, as shown in Figure 3, is properly extended in close proximity of such a contact. Note that this erase scheme is indeed single-cell selective because the substrate contact, common to all the array cells, is kept to the ground.

**Figure 7:**
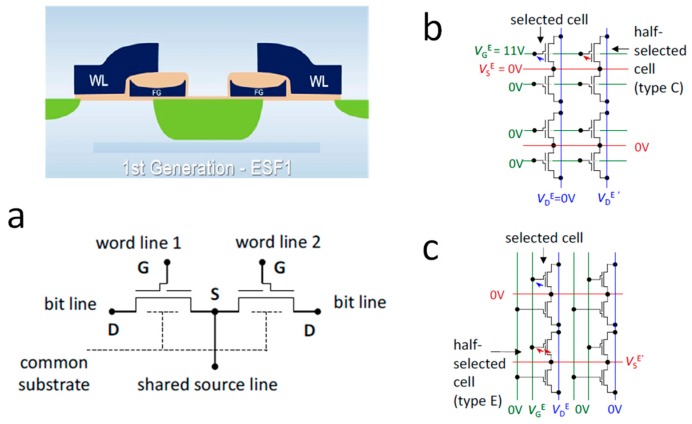
(**a**) Schematic cross-section of the Silicon Storage Technology (SST) cell structure (**top**) and its equivalent circuit (**bottom**) and NOR array with (**b**) classic and (**c**) modified routing together with the respective erase protocol. Reprinted with permission from [53]. Copyright 2015, IEEE.

Although, recently, some more effort was devoted to build new custom synaptic devices and test them in SNNs [49,50,51], a more convincing proof of the feasibility of the floating-gate transistor to build large-scale neuromorphic systems comes from a different approach. The basic idea consists in slightly modifying the routing of commercially available NOR Flash memory arrays to enable a single-cell selective erase operation while keeping the cell structure unchanged. For this purpose, NOR memory arrays developed with a 180 nm technology by Silicon Storage Technology, Inc. (SST) [52] are chosen in refs. [53,54,55,56]. The basic memory cell, as depicted in Figure 7a, features a highly asymmetric structure presenting a floating gate only near the source side, with the gate stack at the drain side made only of the tunneling oxide. In spite of this structure, the program operation can still be performed by CHEI at the source side; as for the erase operation, instead, a positive voltage is applied between the gate and source, resulting in the emission of stored electrons toward the gate by FN tunneling.

The arrangement of such SST cells to make a NOR array is shown in Figure 7b, where the erase voltages are highlighted too. Since both WLs and SLs run parallel to each other and orthogonal to the BLs, the erase protocol involves all the cells in a row at the same time. For this reason, in refs. [54], a modification to the array routing as reported in Figure 7c is proposed, with the WLs now running parallel to the BLs. In this way, single-cell selectivity is achieved during both the program (involving WL, BL, and SL) and erase (involving WL and SL only).

**Figure 8:**
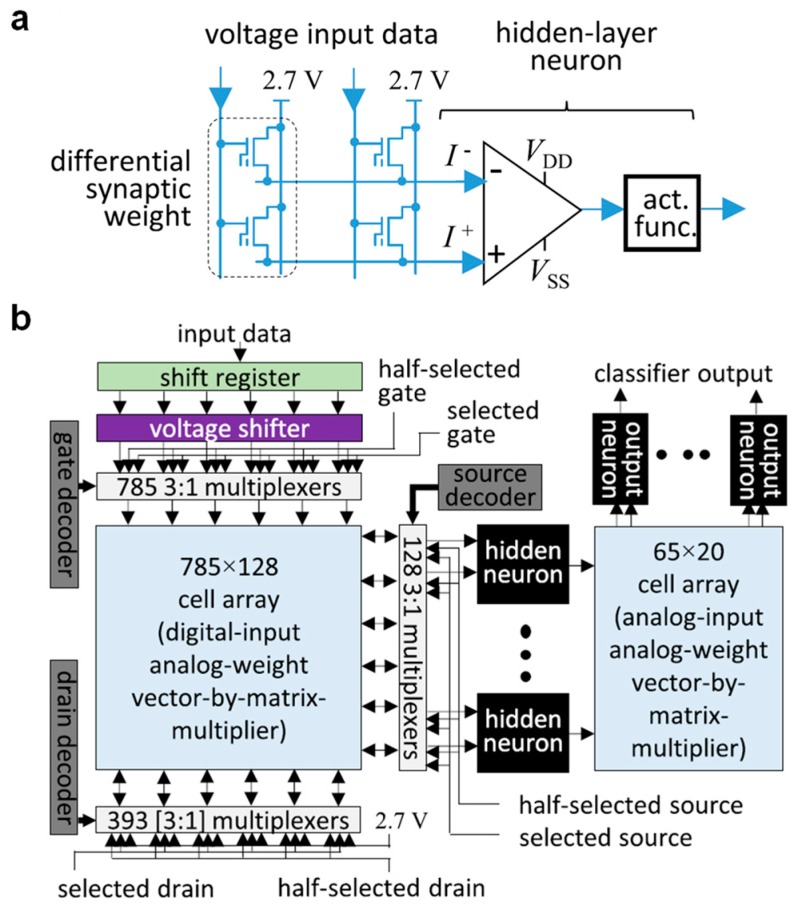
(**a**) Differential implementation of a synaptic connection followed by a hidden-layer neuron, consisting of a differential summing operational amplifier and an activation-function block. (**b**) High-level architecture of the artificial neural network and needed additional circuitry. Reprinted with permission from [55]. Copyright 2018, IEEE.

In refs. [54,55], two SST NOR arrays, re-routed as explained before, are employed to build and test a fully integrated three-layer (784 × 64 × 10) ANN, trained offline on the Modified National Institute of Standards and Technology (MNIST) database for handwritten digit recognition via the backpropagation algorithm [21,22,23]. In particular, in order to enable the implementation of negative weights, and also to reduce random drifts and temperature sensitivity, a differential solution is adopted. As shown in Figure 8a, following this approach, each couple of adjacent memory cells implements a synaptic weight, with the resulting BL currents summed and read by CMOS artificial neurons built exploiting a differential current operational amplifier. The whole one-chip integrated network, whose schematic structure, including two synaptic arrays together with two neuron layers and some additional circuitry, is reported in Figure 8b, has shown a 94.7% classification fidelity with one-pattern classification time and energy equal to 1 µs and less than 20 nJ, respectively. Moreover, a reduction of the total chip active area, amounting to 1 mm^2^ in the discussed work, is expected together with an increase of its performance when moving to the next 55 nm SST technology. In this regard, some preliminary results about MVM were already presented in ref. [56].

**Figure 9:**
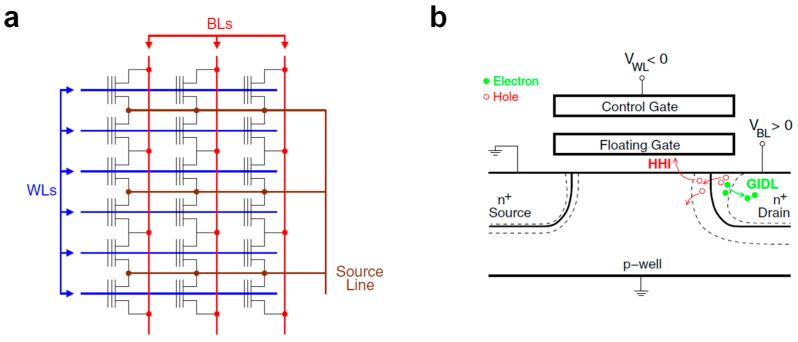
(**a**) Schematic for a mainstream common-ground NOR Flash array and (**b**) proposed physical mechanism exploited for the erase operations. Reprinted with permission from [57]. Copyright 2018, IEEE.

Although this solution based on re-routing commercially available NOR arrays appears promising, it comes together with its main drawback consisting in the increased area occupancy (the single-cell area in the modified array is 2.3 times larger than the original one). A different approach aiming at avoiding this disadvantage is proposed in [57,58,59]. Here, the authors suggest a modified working scheme for a mainstream double-polysilicon common-ground NOR Flash arrays developed in a 40 nm embedded technology by STMicroelectronics (Figure 9a) without any change needed in the cell or array design. While keeping CHEI as the physical mechanism for the program, single-cell selectivity during the erase is achieved by employing hot-hole injection (HHI) in the cell floating gate. In particular, by keeping the source and substrate contacts to the ground while applying a positive and negative voltage to the drain and to the gate, respectively, the developed electric field triggers the generation of holes by band-to-band tunneling at the drain side and accelerates them (Figure 9b); if the applied voltages are high enough, the energy acquired by the holes allows them to overcome the energetic barrier of the tunnel oxide and to be redirected toward the floating gate thanks to the negative gate voltage.

**Figure 10:**
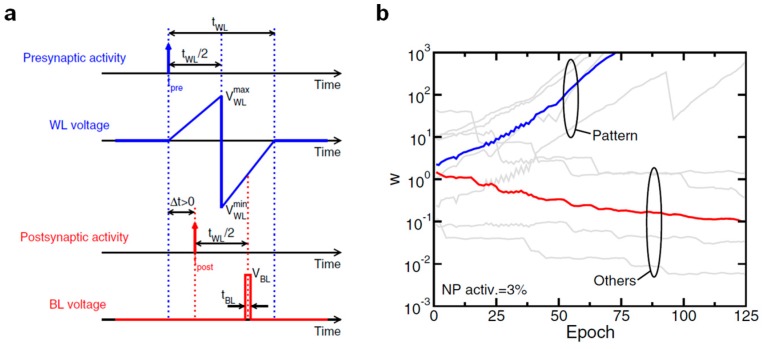
(**a**) Pulse scheme proposed to implement the spike-timing-dependent plasticity (STDP) waveform exploiting the erase mechanism shown in Figure 9b and (**b**) evolution of the weights of the implemented NOR Flash-based spiking neural network during the learning phase. Reprinted with permission from [57]. Copyright 2018, IEEE.

To validate this program/erase scheme in a brain-inspired neural network, the authors demonstrated long-term potentiation/depression through the design of the presynaptic and postsynaptic waveforms as shown in Figure 10a. The short rectangular pulse applied to the BL as a consequence of a postsynaptic fire event overlaps with a positive or negative WL voltage according to the time distance between the presynaptic and postsynaptic spike, Δt. In particular, Δt > 0 leads to long-term potentiation by HHI and Δt < 0 leads to long-term depression by CHEI. To further confirm the validity of this protocol, a prototype two layers 8 × 1 SNN was tested on pattern recognition, producing encouraging results as shown in Figure 10b; in fact, as expected, while the synapses corresponding to the input pattern are quickly potentiated, the remaining ones are gradually depressed.

A final remark, being of great relevance especially in DNN inference, is the finite tuning precision of the cells array, $V_{T}$, and its stability after the offline training phase. In the case of ANN based on NOR Flash memory arrays, two of the most relevant physical mechanisms causing reliability issues of this kind are program noise (PN), determining an inherent uncertainty during the program phase due to the statistical nature of electron injection in the floating gate, and random telegraph noise (RTN), inducing $V_{T}$ instabilities arising from the capture and release of charge carriers in tunnel-oxide defects. In ref. [60], the authors assess the impact of both PN and RTN on a neuromorphic digit classifier through parametric Monte-Carlo simulations. The main result, relevant in terms of projection of the previously discussed results on future technological nodes, is that such non-idealities play a non-negligible role, setting a stringent requirement both on the maximum scalability of the array cell and on the adopted program/erase schemes.

## 4. Memristive Technologies

To replicate neural networks in hardware, memristive devices have been recently investigated for the realization of compact circuits capable of emulating neuron and synapse functionalities. Increasing interest toward these novel device concepts first results from their ability to store information at the nanoscale in an analogue and non-volatile way. Also, they allow the memory to be combined with the computing function, enabling in-situ data processing, also referred to as in-memory computing [11], which is currently the major approach toward the achievement of energy-efficient computing paradigms beyond the von Neumann bottleneck. In detail, the landscape of memristive technologies can be divided into the classes of memristors with two or three terminals, which are explained in the following subsections.

### 4.1. Memristive Devices with 2-Terminal Structure

**Figure 11:**
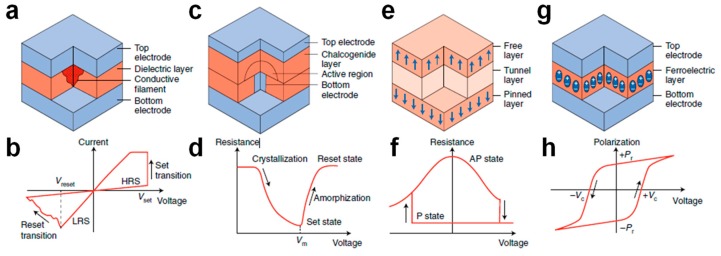
Sketch of the most promising two-terminal memristive devices used in neuromorphic computing applications. (**a**) Structure of resistive switching random access memory (RRAM) device where the insulating switching layer is sandwiched between two metal electrodes. (**b**) Current-voltage characteristics of RRAM displaying that the application of a positive voltage causes an abrupt resistance transition, called set, leading the device from the high resistance state (HRS) to the low resistance state (LRS) while the application of a negative voltage causes a more gradual resistance transition, called reset, leading the device from LRS to HRS. (**c**) Structure of phase change memory (PCM) device where a chalcogenide active layer is sandwiched between two metal electrodes. (**d**) Resistance-voltage characteristics of PCM displaying that the crystallization process in the active layer gradually leading the PCM from HRS to LRS is achieved at voltages below the melting voltage, V_m_, while the amorphization process gradually leading the PCM from LRS to HRS is achieved at voltages above V_m_. (**e**) Structure of spin-transfer torque magnetic random access memory (STT-MRAM) device, where a tunnel layer is sandwiched between two ferromagnetic metal electrodes. (**f**) Resistance-voltage characteristics of STT-MRAM displaying two binary resistance transitions leading the device from the anti-parallel (AP) to the parallel (P) state (set) at positive voltage and from P to AP (reset) at negative voltage. (**g**) Structure of ferroelectric random access memory (FeRAM) device, where a ferroelectric layer is sandwiched between two metal electrodes. (**h**) Polarization-voltage characteristics displaying binary operation between two states with a positive residual polarization, +P_r_, and a negative residual polarization, −P_r_, achieved by application of a positive and negative voltage, respectively. Reprinted with permission from [11]. Copyright 2018, Springer Nature.

As shown in Figure 11, the class of memristive devices with a two-terminal structure covers various physical concepts, such as resistive switching random access memory (RRAM), phase change memory (PCM), spin-transfer torque magnetic random access memory (STT-MRAM), and ferroelectric random access memory (FeRAM), which share a very simple structure consisting of a metal-insulator-metal (MIM) stack, where an insulating layer is sandwiched between two metallic electrodes called the top electrode (TE) and bottom electrode (BE), respectively. As a voltage pulse is applied, these devices undergo a change of physical properties of the material used as the switching layer, which results in a change of the resistance for RRAM and PCM, magnetic polarization for STT-MRAM, and electrical polarization for FeRAM. Importantly, all these memristive elements offer the opportunity to read, write, and erase the information in memory states by electrical operations on the device, thus making them potentially more attractive in terms of scalability than other memory concepts, such as the Flash memories based on charge storage.

Figure 11a shows the MIM stack of the RRAM device, where an insulating oxide material serves as the switching layer [61,62,63]. To initiate the device, a preliminary electrical operation called forming is performed by application of a positive voltage at TE by causing a soft breakdown process, leading to the creation of a high conductivity path containing oxygen vacancies and/or metallic impurities, also known as a conductive filament (CF), within the oxide layer. This results in the change of the resistance of the device from the initial high resistance state (HRS) to the low resistance state (LRS). After forming, in the case of bipolar RRAM devices, the application of negative/positive voltage pulses at TE leads the device to experience reset and set transitions, respectively. The application of a negative pulse causes the rupture of CF (reset process), leading to the opening of a depleted gap via drift/diffusion migration of ion defects from BE to TE, hence to the HRS. On the other hand, the application of a positive pulse allows the gap to be filled via field-driven migration of ion defects from TE to BE, thus leading the device back to LRS (set process) [64,65]. Two resistance transitions can be noted by the current-voltage characteristics shown in Figure 11b, which evidence both the abrupt nature of the set process due to the positive feedback loop involving the two driving forces for ion migration, namely the electric field and temperature, and the more gradual dynamics of the reset process due to the negative feedback occurring within the device as a negative pulse is applied [66]. Similar to the bipolar RRAM described in Figure 11b, which typically relies on switching layers, including HfO_x_ [67], TaO_x_ [68], TiO_x_ [69], SiO_x_ [70], and WO_x_ [71], the conductive-bridge random access memory (CBRAM), where metallic CFs are created/disrupted between active Cu/Ag electrodes, has also received strong interest in recent years [72]. In addition to bipolar RRAM concepts, another type of filamentary RRAM called unipolar RRAM, typically based on NiO [73,74,75], has been widely investigated, evidencing that pulses with the same polarity can induce both set and reset processes as a result of the key role played by Joule heating for the creation/disruption of CF [73,75]. Moreover, the RRAM concept also includes non-filamentary devices referred to as uniform RRAM, exhibiting an interface resistive switching due to the uniform change of a Schottky or tunneling barrier on the whole cell area [76]. One of the fundamental features making RRAM suitable for in-memory computing is the opportunity to modulate its resistance in an analog way, thus enabling multilevel operation via the storage of at least 3 bit [77,78,79,80,81]. In addition to multilevel operation, it also combines high scalability up to 10 nm in size [82] and the opportunity to achieve 3D integration [83].

Figure 11c shows the schematic structure of a PCM device, which relies on a chalcogenide material, such as Ge_2_Sb_2_Te_5_ (GST) [84], as the switching layer. Here, resistance variation arises from an atomic configuration change within the active layer from the crystalline to the amorphous phase and vice-versa via application of unipolar voltage pulses at TE [85,86,87]. As a voltage higher than the voltage, V_m_, needed to induce the melting process within the active layer is applied across the cell, local melting takes place within the chalcogenide material, leading the device to HRS as a result of the pinning of the Fermi level at the midgap. Otherwise, if the applied voltage is below V_m_, a gradual crystallization process is triggered via local Joule heating, leading PCM to LRS [88]. These physical processes can be better visualized by the resistance-voltage characteristics in Figure 11d, where the set transition displays a gradual behavior due to the gradual crystallization process induced by Joule heating while the reset transition displays faster dynamics than the set transition. Compared to RRAM, where the HRS/LRS ratio is about 10, PCM offers a higher resistance window, ranging from 100 to 1000, which makes PCM very attractive for multilevel operation as reported in [89], where a 3 bits/cell PCM device was demonstrated. Moreover, in addition to classic GST, other materials, such as GeSb [90], doped In-Ge-Te [91], and Ge-rich GST [92], have been investigated, receiving strong interest since they offer higher crystallization temperatures for enhanced retention performances.

Figure 11e shows the schematic structure of an STT-MRAM device based on an MIM stack called magnetic tunnel junction (MTJ), including an ultrathin tunneling layer (TL), typically in MgO, interposed between two ferromagnetic (FM) metal electrodes, typically in CoFeB, called the pinned layer (PL) and free layer (FL), respectively [93,94,95]. Unlike RRAM and PCM enabling multilevel operation, STT-MRAM allows only two states to be stored, with a very small resistance window of the order of a factor 2 [94] because of the tunnel magneto-resistance (TMR) effect [96]. The two states are encoded in the relative orientation between PL magnetic polarization, which is fixed, and FL magnetic polarization, which is instead free to change via the spin-transfer torque physical mechanism discovered by Slonczewski [97] and Berger [98] in 1996. As a positive voltage is applied at TE, a current of electrons with the same spin-polarization of the fixed layer is transmitted through the tunneling layer, causing the transition of the polarization orientation from anti-parallel (AP) to parallel (P), which leads the device to LRS. In contrast, as a negative bias is applied, the reflection back of electrons entering from the free layer with the opposite magnetization takes place, thus causing the transition from the P to AP state, hence from LRS to HRS. Figure 11f shows the resistance response of the STT-MRAM device as a function of the applied voltage, evidencing that the application of positive/negative voltage pulse induces set/reset transition with very abrupt dynamics, which further supports the incompatibility of STT-MRAM with multilevel applications. However, STT-MRAM has shown high potential in scalability, as reported in ref. [99], fast switching speed [100], and almost unlimited cycling endurance [101,102].

Figure 11g shows the MIM stack of FeRAM, where an insulating layer based on a ferroelectric (FE) material, typically in doped HfO_2_ [103] or perovskite materials [104,105], is sandwiched between two metal electrodes. Its operation principle relies on the polarization switching within the FE layer due to the rotation of electrical dipoles under an external bias [106]. As shown by the polarization-voltage characteristics in Figure 11h, a positive voltage above the coercive voltage, +V_c_, at TE induces the set transition, leading the device to exhibit a positive residual polarization, +P_r_, whereas a voltage more negative than −V_c_ leads the device to exhibit a negative residual polarization, −P_r_. Importantly, note that the FE switching process does not impact on the device resistance, which makes FeRAM unusable as resistive memory.

### 4.2. Memristive Devices with Three-Terminal Structure

**Figure 12:**
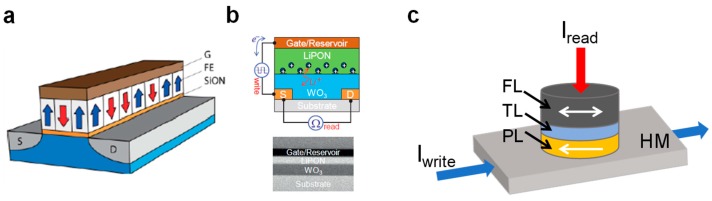
Sketch of three fundamental examples of three-terminal memristive devices. (**a**) Schematic structure of ferroelectric field-effect transistor (FeFET) device, where the ferroelectric switching phenomenon allows the transistor threshold voltage to be modulated, thus gradually changing the channel conductivity. (**b**) Schematic structure of electro-chemical random access memory (ECRAM) device, where the channel conductivity is controlled by the migration of ion species, e.g., Li^+^ ions, into an electrolyte material being induced by the voltage applied at the gate terminal. (**c**) Schematic structure of spin-orbit torque magnetic random access memory (SOT-MRAM), where the current flow in a heavy metal (HM) line causes a polarization switching in the MTJ-free layer, resulting in a device conductance change. Reprinted with permission from [107,108]. Copyright 2017, IEEE. Copyright 2018, IEEE.

In addition to the two-terminal devices, memristive concepts also include the class of three-terminal devices whose main examples are those depicted in Figure 12, namely (a) the ferroelectric field-effect transistor (FeFET) [107], (b) the electro-chemical random access memory (ECRAM) [108], and (c) the spin-orbit torque magnetic random access memory (SOT-MRAM) [109]. Other interesting three-terminal concepts that have been recently investigated for neuromorphic computing applications are the 2D semiconductor-based mem-transistors [110,111] and the domain-wall-based magnetic memories [112,113].

Figure 12a shows the structure of the FeFET consisting of an MOS transistor with an FE material, such as doped-HfO_2_ [103], and perovskites [106], serving as the gate dielectric. Here, the application of external pulses at the gate terminal induces a non-volatile polarization switching within the FE dielectric, leading to a change of the transistor threshold, hence of the channel conductivity, which can be probed simply by reading the current at the drain terminal. As a result, the FeFET concept allows significant issues due to transient read currents and destructive read operation limiting FeRAM operation to be overcome. This three-terminal device has recently been operated into memory arrays with 28 nm CMOS technology [114] and exhibits a strong potential for the development of 3D structures [115]. Also, it has been operated to replicate synapse [116] and neuron [117,118] functions, which, combined with 3D integration opportunity, makes it a strong candidate for neuromorphic computing applications.

Figure 12b illustrates the device structure of the ECRAM consisting of an MOS transistor where a solid-state electrolyte based on inorganic materials, such as lithium phosphorous oxynitride (LiPON) [108,119], or organic materials, such as poly (3, 4-ethylenedioxythiophene):polystyrene sulfonate (PEDOT:PSS) [120], is used as the gate dielectric. Its operation relies on the intercalation/de-intercalation of ions in a channel layer to tune the device conductance. As reported in [108], the intercalation of Li^+^ ions into the WO_3_ layer by application of a positive voltage at the gate terminal leads the device to experience a conductance increase whereas the de-intercalation of Li^+^ ions under negative bias leads the device to experience a conductance decrease. The linear conductance change is achievable in ECRAM thanks to the decoupling of read/write paths, which makes this device concept very attractive for synaptic applications, mainly for hardware implementation of synaptic weights in ANNs, where analog and symmetric weight updates play a crucial role. Also, the device investigated in [108] provides fast operation at the nanosecond timescale, thus opening the way toward a significant acceleration of the training process in hardware ANNs.

Figure 12c shows the device structure of the SOT-MRAM, where a heavy metal (HM) line, typically in Pt [121] or Ta [122], is located under an MTJ. This three-terminal device is programmed by the flow of a horizontal current through the HM line, which induces a spin accumulation as a result of the spin Hall or the Rashba effects [123,124], leading to the switching of magnetic polarization in the MTJ FL. Unlike the program operation, the read operation can be performed by measuring the vertical current flowing in MTJ as a result of the TMR effect, which means that the three-terminal structure of SOT-MRAM offers the opportunity to decouple read/write current paths and consequently improve the endurance performance compared with STT-MRAM. Regarding device applications, SOT-MRAM was used to implement neuromorphic computing in ANNs, by exhibiting the synapse function [125], the neuron function [126], and the associative memory operation [127].

## 5. Memristive Neuromorphic Networks

Thanks to their rich physics and nanoscale size, memristive concepts are believed to be promising candidates to achieve the huge density and behavior of real synapses and neurons, thus enabling brain-like cognitive capabilities in hardware neural networks. Based on this appealing approach, many hardware or mixed hardware/simulation implementations of the neural networks currently dominating the neuromorphic computing scenario, namely the DNNs and the SNNs, have been proposed.

### 5.1. DNNs with Memristive Synapses

DNNs encompass various ANN architectures, such as feedforward MLP and convolutional neural network (CNN) [36], that have attracted wide interest in the neuromorphic computing scenario thanks to the excellent performance achieved in machine learning tasks, such as image classification [128], face verification [129], and speech recognition [130]. Because of the very high complexity of the CNN architecture, which consists of a deep hierarchy of convolutional layers followed by some fully connected layers, and processing strategy, which is based on the extraction of the most significant features of submitted images via the application of large sets of filters, hardware implementation of DNN tasks with memory devices has mostly been focused on feedforward MLP networks. In this type of ANN, the training phase is based on a supervised learning algorithm called backpropagation [21,22,23] and consists of three sub-procedures called forward propagation, backward propagation, and weight update [36]. Note that although the backpropagation algorithm is chiefly considered lacking in biological plausibility [131], recent works have questioned this aspect [132]. During training, upon any input presentation from a training database containing images of objects, digits, or faces, the input signal propagates in the forward direction from the input to output layer, passing through the multiplication by synaptic weights of each layer and the summation at the input of each hidden/output neuron. Forward propagation yields an output signal, which is compared with the target response of the network, namely the label of the submitted image, thus leading to the calculation of the corresponding error signal. At this point, the calculated error signal is propagated in the backward direction from the output to the input layer and is used to update all the synaptic weights, hence the name backpropagation. Repeating this scheme for every image of the training database for a certain number of presentation cycles or epochs, the optimization of synaptic weights is achieved, leading the network to specialize on the training database. After, the training phase is followed by the test phase, namely the phase where the classification ability of DNN is evaluated by submitting another database, called the test dataset, only once, via forward propagation of the signal encoded in all the test examples [36].

The downside of the outstanding results achieved running DNNs in software on high-performance digital computers, such as GPU and TPU, or very large servers is given by the excessive power consumption and latency due to the von Neumann architecture. To overcome this issue, memristive devices, in particular RRAM and PCM, have been intensively investigated to accelerate artificial intelligence (AI) applications in hardware thanks to their ability to execute in-memory computing with extremely high energy efficiency and speed by exploiting basic physical laws, such as the Ohm’s law and Kirchhoff’s law [11]. However, hardware implementation of a real in-situ weight update for DNN training has been challenged by critical non-idealities affecting the conductance response of the majority of memristive devices, mainly RRAM and PCM, during set (potentiation) and reset (depression) processes, such as the non-linearity, the asymmetry, and the stochasticity [34,133,134]. Motivated by these significant limitations, a wide range of alternative materials and technologies have been intensively investigated, leading to the recent emergence of novel concepts, such as ECRAM [108] and the ionic floating gate [135], thanks to their highly linear, symmetric, and analog conductance behavior.

**Figure 13:**
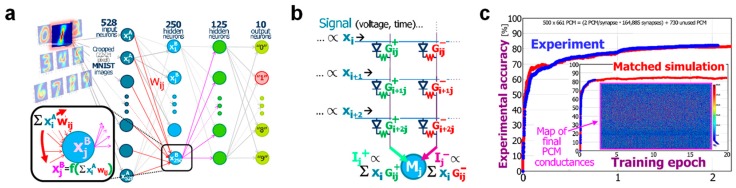
(**a**) Schematic representation of a three-layer DNN operated on the MNIST database for an image classification task. (**b**) Weight implementation in DNN by differential pairs of 1T1R PCM cells with conductances G_ij_^+^ and G_ij_^−^, which provide a positive current and a negative current, respectively. (**c**) Experimental classification accuracy achieved by three-layer DNN during the inference phase. Reprinted with permission from [12]. Copyright 2014, IEEE. Deep neural networks, DNNs; Modified National Institute of Standards and Technology, MNIST; one-transistor-one-resistor, 1T1R.

In the last 10 years, great advances in crossbar-based demonstrations of DNNs for pattern classification have been achieved using RRAM and PCM devices [12,13,136,137,138]. In ref. [12], a medium-scale crossbar array containing 165,000 PCM devices with a one-transistor-one-resistor (1T1R) structure was used to demonstrate an image classification task by hardware implementation of the three-layer DNN schematically shown in Figure 13a. This network is based on an input layer with 528 input neurons, a first hidden layer with 250 neurons, a second hidden layer with 125 neurons, and an output layer with 10 neurons, and was operated on a cropped version (22 × 24 pixels) of handwritten digit images from the MNIST database for training and test operations. To implement positive and negative synaptic weights of the network, Burr et al. proposed a differential configuration based on pairs of 1T1R PCM cells with conductance, G^+^ and G^-^, respectively, as shown in Figure 13b. According to this structure, each weight can be potentiated or depressed by increasing G^+^ with fixed G- or increasing G- with fixed G^+^, respectively. Also, the network was implemented with software neurons, providing the conversion of the sum of input currents into an output voltage by application of the tanh non-linear function. After the training process, which was carried out on 5000 MNIST images by using a complex pulse overlap scheme, the network’s classification ability was evaluated, leading to a best performance of only 83% due to the asymmetry and non-linearity of the PCM G-response (Figure 13c). To tackle this limitation, a novel artificial synapse combining the 1T1R differential pair with a three-transistor/one-capacitor (3T1C) analog device was presented in ref. [138]. This led the PCM-based DNNs with improved hardware synapses to match the software performance on both the MNIST and CIFAR databases [139]. Later, other DNN implementations in small-scale 1T1R RRAM crossbar arrays were demonstrated, enabling MNIST classification with 92% test performance [137] and gray-scale face classification on the Yale face database with 91.5% performance [136], thanks to the RRAM conductance responses displaying high linearity and symmetry in both update directions. Moreover, an alternative approach aiming at combining high performance with high energy efficiency was proposed in ref. [140]. Here, after an off-line training resulting in the optimization of synaptic weights in the software, the floating-point accuracy of synaptic weights was reduced only to five levels, which were stored in a hardware 4 kbit HfO_2_ RRAM array using a novel multilevel programming scheme. The following execution of the inference phase with the experimental conductances stored into the 4 kbit RRAM array led to a maximum classification accuracy of 83%. A simulation-based study showed that the implementation of synaptic weights using more conductance levels can move performance beyond 90% with larger arrays.

### 5.2. SNNs with Memristive Synapses

Although DNNs have shown to be capable of excellent performance in fundamental cognitive functions, exceeding the human ability in some cases [128,141], the interest in SNNs is rapidly increasing thanks to their attempt to replicate structure and operation principles of the most efficient computing machine found in nature, which is the biological brain. The brain can efficiently learn, recognize, and infer in an unsupervised way thanks to the plasticity of biological synapses controlled by local rules, such as STDP, which has recently inspired many hardware implementations of synaptic plasticity at the device and network level exploiting the attractive physical properties of memristive devices.

**Figure 14:**
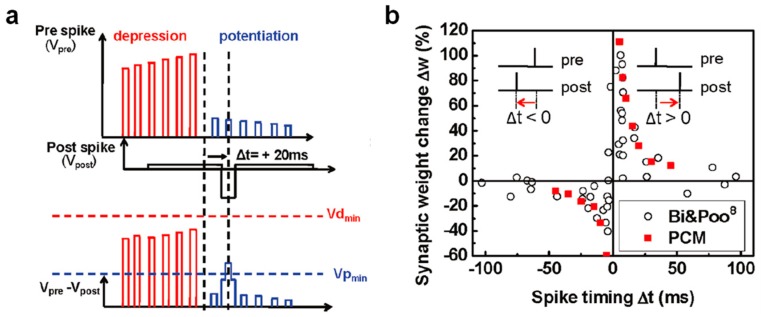
(**a**) PRE and POST spike waveforms applied at terminals of a PCM-based synaptic device to change its weight via an overlap-based STDP scheme. The application of a positive time delay of 20 ms leads to a conductance increase (potentiation) in the PCM synapse since the spike overlap leads the effective voltage across the PCM to cross the potentiation threshold whereas the higher depression threshold is not hit. (**b**) Measured weight change as a function of the spike timing achieved using a PCM synapse against experimental data collected by Bi and Poo in biological synapses. Reprinted with permission from [144]. Copyright 2012, American Chemical Society.

One of the earliest STDP demonstrations at the memristive device level was performed by Jo and coauthors in ref. [142] by using an Ag/Si-based CBRAM device as the synapse and a time-division multiplexing approach based on synchronous time frames which was designed to achieve STDP characteristics thanks to the conversion of the time delay into the amplitude of the pulse to be applied across the synaptic device. After this precursor implementation, another scheme based on voltage overlap at the terminals of memristive synapses was experimentally demonstrated in both RRAM [143] and PCM [144]. Both works demonstrate potentiation and depression characteristics very close to biological STDP, exploiting the analog modulation of device conductance achieved via the superposition of voltage spikes with suitably tailored waveforms. Specifically, Kuzum et al. proposed the voltage waveforms shown in Figure 14a as PRE and POST spikes for achieving potentiation in PCM devices [144]. As the relative delay is positive, in this case Δt = 20 ms, the overlap of the PRE spike, which consists of a sequence of high positive pulses with increasing amplitudes followed by another sequence of small positive pulse with decreasing amplitudes, with the POST spike, which consists of a single 8 ms long negative pulse, leads the total voltage across the PCM cell, V_pre_ − V_post_, to only cross the minimum threshold for potentiation, v_P_, thus leading the synapse to undergo potentiation via a set process within PCM. Changing the sign of Δt, depression was also demonstrated, thus allowing the STDP characteristics shown in Figure 14b to be achieved, which exhibit a very nice agreement with the Bi and Poo measurements [38]. Moreover, note that this scheme offers the opportunity to finely tune the shape of STDP characteristics, by suitably designing the PRE spike waveform [144]. Taking inspiration from this approach based on overlapping spikes across the memristive device, more recently, other significant STDP demonstrations were achieved in individual two-terminal memristive devices, thus enabling unsupervised learning in small-scale memristive SNNs [145,146,147,148,149]. However, the synapse implementation using individual two-terminal memristive devices might suffer from serious issues, such as (i) the requirement to control the current during set transition in RRAM devices to avoid an uncontrollable CF growth [64], which would reduce the synapse reliability during potentiation; (ii) the sneak paths challenging the operation of crossbar arrays; and (iii) the high energy consumption.

**Figure 15:**
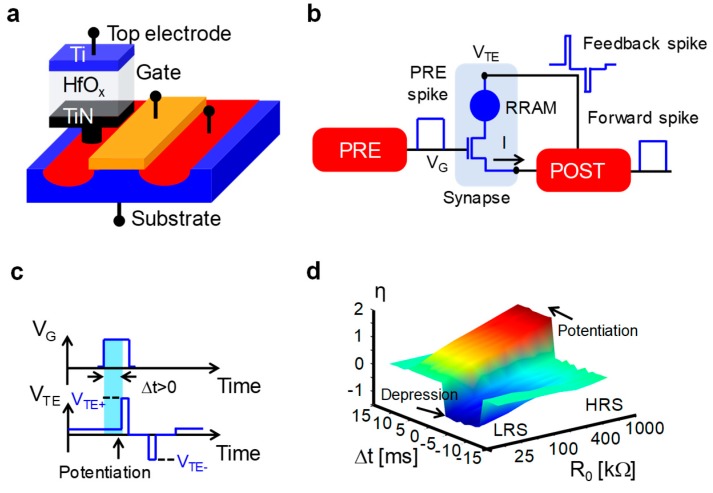
(**a**) Schematic structure of the 1T1R RRAM structure. (**b**) Schematic representation of the 1T1R structure as a synapse to achieve STDP in hardware via overlapping PRE and POST voltage spikes applied at the gate terminal and RRAM top electrode, respectively. (**c**) Schematic sketch of PRE and POST overlapping spikes leading to synapse potentiation via the activation of a set process in the RRAM cell. (**d**) STDP characteristics experimentally demonstrated in the 1T1R RRAM synapse. Adapted with permission from [151,152]. Copyright 2016, IEEE.

To overcome these drawbacks, a novel hybrid CMOS/memristive STDP synapse using the 1T1R structure was proposed in refs. [150,151]. Figure 15a shows the schematic structure of the 1T1R device presented in ref. [151], where a Ti/HfO_x_/TiN RRAM is serially connected to the drain of an MOS transistor acting as selector and current limiter. As schematically shown in Figure 15b, the ability of the 1T1R cell to operate as a synapse capable of STDP was validated in the hardware [152]. The 1T1R synapse operation can be explained as follows. The application of a pulse designed as a PRE spike at the gate terminal of the transistor combined with the low voltage bias applied at the TE of the RRAM device activates a current flowing toward the BE. At this point, the current enters in an integrate-and-fire circuit implementing POST where it is integrated, causing an increase of the POST internal potential, V_int_. As a sequence of PRE spikes leads the POST to cross its internal threshold, the POST emits both a forward spike toward the next neuron layer and a suitably designed spike, including a positive pulse followed by a negative pulse, being delivered at TE, thus creating the conditions for synaptic weight update according to STDP [151]. As shown in Figure 15c, if the PRE spike anticipates the POST spikes (Δt > 0), only the positive pulse of the POST spike with amplitude V_TE+_ (V_TE+_ > V_set_) overlaps with the PRE spike, thus inducing a set transition within the RRAM device, leading RRAM to LRS, and, therefore, the synapse to be potentiated. Otherwise, if the PRE spike follows the POST spike (Δt < 0), only the negative pulse with amplitude V_TE−_ (|V_TE−_| > |V_reset_|) overlaps with the PRE spike, thus inducing a reset transition within the RRAM device, leading RRAM to HRS, and, therefore, the synapse to be depressed (not shown). Thanks to this operation principle, the 1T1R synapse was shown to capture STDP functionality implementing the 3D characteristics shown in Figure 15d, where the relative change in conductance, η = log_10_(R_0_/R), is plotted as a function of the initial resistance state, R_0_, and relative delay, Δt. They support potentiation/depression at positive/negative Δt, evidencing that maximum potentiation is obtained for R_0_ = HRS, whereas maximum depression is obtained for R_0_ = LRS. If the 1T1R synapse is initially in LRS/HRS, no potentiation/depression occurs because it cannot overcome the boundary conductance values set by LRS and HRS [151,152,153]. Importantly, note that the weight change in the 1T1R synapse can be induced only via spike overlap, hence only for delays in the range −10 ms < Δt < 10 ms in this experiment [152].

Although the STDP characteristics achieved in the 1T1R RRAM synapse [151,152] display a squared shape due to binary operation of the RRAM cell instead of the exponentially decaying behavior observed in biological experiments, the plasticity of the 1T1R synapse was exploited in many SNN implementations enabling neuromorphic tasks, such as unsupervised learning of space/spatiotemporal patterns [151,152,154,155], the extraction of auditory/visual patterns [156,157], pattern classification [158,159,160], and associative memory [161,162,163], in both simulation and hardware.

**Figure 16:**
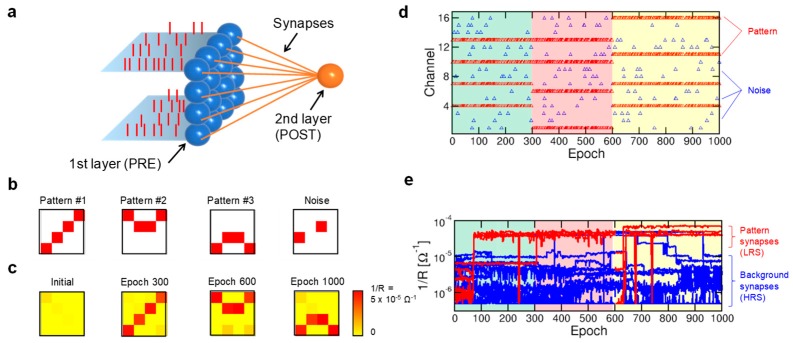
(**a**) Schematic sketch of a single-layer perceptron network where a 4 x 4 input layer is fully connected to a single POST. (**b**) Sequence of three visual patterns (Pattern #1, Pattern #2, and Pattern #3) submitted to the neural network during training process and an example of a random noise image, which is alternatively applied to patterns according to a stochastic approach. (**c**) Conductance/weight color plots measured at epochs 0, 300, 600, and 1000 evidencing the ability of the synaptic weights to adapt to submitted patterns thanks to selective potentiation of pattern synapses and noise-induced depression of background synapses. (**d**) Raster plot of PRE spikes applied to pattern and background input channels during the learning experiment. (**e**) Time evolution of the measured synaptic conductance during three phases of the unsupervised learning experiment showing convergence of pattern/background synapses to LRS/HRS. Reprinted from [152].

Figure 16a shows the schematic representation of the RRAM-based SNN used in ref. [152] to demonstrate unsupervised learning of visual patterns in hardware. This perceptron SNN consists of 16 PREs connected to a single POST via individual synapses with the 1T1R RRAM structure of Figure 15a. Pattern learning experiment is based on three sequential phases where only one 4 × 4 visual pattern among Pattern #1, Pattern #2, and Pattern #3 shown in Figure 16b is submitted to the input layer, and was conducted using a stochastic approach according to which the probability to submit the pattern image or a random noise image similar to the last 4 × 4 pattern in Figure 16b at every epoch is 50%. Using this training approach, Figure 16c shows that the submission of three patterns alternated with noise resulted in the on-line adaptation of SNN synapses to the presented pattern in all three phases, evidencing a selective potentiation of synapses within the submitted pattern due to the correlated spiking activity of corresponding PREs and the depression of synapses outside the pattern, typically called background synapses, due to the uncorrelated nature of noise inducing POST spike-PRE spike depression sequences for the background with a high probability [151,152]. Note that the frequency and amount of submitted noise has to be carefully designed to prevent learning dynamics from becoming unstable [164]. To further support the unsupervised pattern learning ability of SNN with 1T1R RRAM synapses, Figure 16d shows the raster plot of spikes generated by PREs during the whole experiment, leading to the time evolution of synaptic conductance evidenced in Figure 16e, where the pattern/background synaptic conductance converges to LRS/HRS at the end of each training phase. Note that the stochastic approach used in this experiment also allowed for the implementation of multiple pattern learning by a winner-take-all scheme [165] based on the use of software inhibitory synapses between 2 POSTs, and unsupervised learning of gray-scale images [152].

The main drawbacks generally limiting the implementation of synaptic plasticity in overlap-based synaptic concepts, such as the 1T1R synapse, are the pulse duration and energy efficiency. Overlap-based implementations first require a pulse width of the order of time delays to allow for conductance change within the device, which results in pulses with a long duration causing a high power consumption. In addition to this, the need for long pulses to program overlap-based memristive devices also causes too slow signal processing in large neuromorphic networks, which leads to low throughput performance [166].

**Figure 17:**
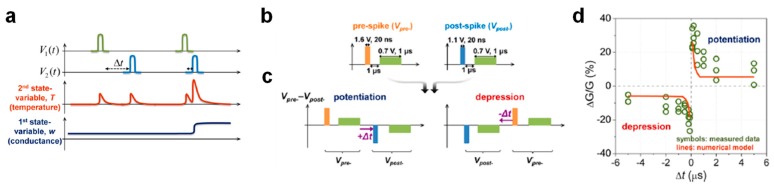
(**a**) Schematic representation of a non-overlap scheme enabling STDP in second-order memristors. Short-term memory effects observed in second-order physical variables, e.g., internal temperature, allow for the implementation of potentiation/depression for short/long delays. (**b**) PRE and POST spike waveforms applied at top electrode (TE) and bottom electrode (BE) to implement non-overlap STDP. (**c**) Effective voltage across a second-order memristor to induce potentiation (**left**) and depression (**right**). (**d**) STDP characteristics measured in a second-order memristor against calculated curves achieved by numerical modeling. Reprinted with permission from [168]. Copyright 2015, American Chemical Society.

An alternative approach to achieve synaptic plasticity overcoming the limitations affecting overlap-based memristive devices consists of the adoption of non-overlap memristive devices, such as the second-order memristor [167,168]. Unlike first-order memristors, such as RRAM and PCM, where device conductance can change only if overlapping voltage pulses are applied at device terminals, resistive switching in second-order memristors can take place by sequential application of two spikes with a certain Δt at device terminals as a result of short-term memory effects encoded in the time evolution of second-order variables, e.g., the internal temperature. As shown in Figure 17a, if Δt is long, two sequential spikes applied at terminals of a second-order memristor induce small independent changes in temperature, which results in no conductance change. On the contrary, if Δt is short, the superposition of the effects of applied spikes results in a large change in temperature thanks to a limited thermal constant of about 500 ns, thus leading to a long-term conductance variation in the device as a result of short-term memory effects. Importantly, short memory effects observed in second-order memristors have recently attracted great interest because they can allow for the emulation in hardware of a fundamental biological process playing a key role in the real synapse response as the Ca^2+^ ion dynamics [169,170] and to finely replicate biological STDP and SRDP [168,171]. An interesting STDP demonstration by a second-order memristor is reported in ref. [168]. Here, a Pt/Ta_2_O_5−x_/TaO_y_/Pd RRAM device was operated as a non-overlap synapse to achieve STDP via sequential application of PRE and POST voltages. As shown in Figure 17b, the PRE spike consists of a positive pulse with amplitude of 1.6 V and duration of 20 ns followed after 1 μs by a longer positive pulse with amplitude of 0.7 V and duration of 1 μs whereas the POST spike includes a positive pulse with amplitude of 1.1 V and duration of 20 ns followed after 1 μs by a longer positive pulse with amplitude of 0.7 V and 1 μs width. Note that both the first pulse, called the programming element, and the second pulse, called the heating element, within PRE and POST spikes cannot cause independently a conductance change in the RRAM device. The application of the PRE/POST spike at TE/BE of the RRAM device results in an effective voltage drop across the device, evidencing a PRE–POST spike sequence for positive Δt and POST–PRE spike sequence for negative Δt, as shown in Figure 17c. In the case of the PRE–POST spike sequence (Δt > 0), the heating effect of the PRE spike affects the POST spike, making the positive change in conductance due to the negative programming pulse in the POST higher than the negative change in conductance due to the positive programming pulse in the PRE, hence causing the non-overlap RRAM synapse to undergo potentiation. On the other hand, in the case of the POST–PRE sequence (Δt < 0), the opposite occurrence order of spikes results in an effective negative conductance change in the Pt/Ta_2_O_5−x_/TaO_y_/Pd RRAM device, resulting in the depression of the non-overlap synapse. Figure 17d shows the STDP characteristics experimentally measured in the Pt/Ta_2_O_5−x_/TaO_y_/Pd RRAM device for variable Δt in the range –6 μs – 6 μs, which exhibit strong similarity with biological data and a good agreement with simulation results achieved by a numerical model of the second-order memristor.

Similar to the second-order memristor device, other memristive concepts also allowed bio-realistic synaptic plasticity to be demonstrated using non-overlap schemes. In ref. [172], an atomic switch RRAM, whose stack includes a silver BE, an Ag_2_S-based solid electrolyte, and a metal TE separated from the Ag_2_S layer by a nanogap, was proposed as an artificial synapse thanks to the short-term memory effects controlling its physical processes. In fact, the application of voltage pulses at TE induces the gradual creation of an Ag atomic bridge within the nanogap leading to a short-term potentiation process after a few pulses, resulting in an incomplete atomic bridge, which is followed by a long-term potentiation process achieved after many pulses resulting in the formation of a complete atomic bridge. In addition to short-term plasticity due to the spontaneous relaxation process of the atomic bridge, this non-overlap device also offers the opportunity to capture SRDP potentiation and depression depending on whether the frequency of the applied pulses is high or low. Thanks to this functionality, the sequential learning of visual patterns was demonstrated in a 7 × 7 array of Ag_2_S inorganic synaptic devices.

Another memristive concept to implement non-overlap synapses in hardware was recently presented in ref. [171]. Here, a hybrid device based on the serial configuration of a volatile RRAM with a SiO_x_N_y_:Ag stack serving as the select device and a non-volatile RRAM serving as the resistive device, also known as a one-selector-one-resistor (1S1R) structure, was designed to demonstrate non-overlap synaptic plasticity for neuromorphic computing. Exploiting spontaneous relaxation of CF similar to the one taking place in atomic switches, the introduction of a volatile RRAM or diffusive memristor in series to a non-volatile RRAM, where conductance change can only be induced by the electric field, enabled 1S1R synapses capable of both SRDP and STDP depending on the rate or occurrence timing of PRE and POST spikes applied in sequence at TE. Note that the strong potential of 1S1R synapses for neuromorphic computing applications was also investigated in simulation in [173,174]. Moreover, diffusive memristors developed in ref. [171] were used as neurons to build in hardware a fully memristive neural network, which was shown to achieve outstanding performance in a pattern classification task by the implementation of unsupervised learning [175].

## 6. Discussion

While neuromorphic networks have recently demonstrated an excellent ability in fundamental cognitive computing applications, such as image classification and speech recognition, their large-scale hardware implementation is still a major challenge. Achieving such a goal primarily requires nanoscale, energy-efficient, and fast devices capable of emulating faithfully high-density, ultra-low power operation and low latency of biological synapses and neurons. Moreover, depending on the architecture (DNN or SNN) and the application of neuromorphic networks, such devices should also fulfill other significant requirements, such as high retention, high linearity in conductance response, and long endurance [35]. In Table 1, the CMOS-based and memristive emerging memory devices investigated for neuromorphic computing we discussed in Section 3 and Section 4 are compared in terms of performance, reliability, and suitability for DNN, with the distinction between training and inference phases, and SNN applications; however, it is evidenced that no emerging memory device can currently optimize all the metrics for any network architecture and application.

To efficiently execute DNN online training in hardware, high speed and low energy consumption are two essential features of synaptic devices to maximize the network throughput, namely the rate of trained patterns, and enable DNNs in embedded systems, respectively. In addition to these features, high accuracy in weight update operation imposes the use of devices exhibiting a conductance response with a high degree of linearity. This functionality makes almost all the emerging devices unsuitable as synaptic devices for online training. The only exception is represented by novel Li-ion devices, which appear to be very promising, with a simulated performance of around 98% [119], even though the necessary technology maturity and high-density integration have not been reached yet. Alternatively, more complex structures, including multiple pair of memristive devices, such as PCM and RRAM, could mitigate the need for high linearity, but at the expense of a lower integration density [176].

Differently from DNN online training consisting of forward propagation, backpropagation, and weight update operations, DNN inference only relies on forward propagation, which means that the high linearity needed to accurately update the weights is not an essential feature of synaptic devices for this task. Specifically, hardware suitable for optimizing the inference process should primarily exhibit low latency to accelerate the classification of each test pattern and low-power consumption to enable DNN inference at the edge. In addition to these features, high retention of analogue states is also essential to prevent charge fluctuations in CMOS devices [177], stochastic noise in RRAM [178], and resistance drift in PCM [179] from degrading the weights programmed in one shot after the off-line training procedure. These requirements can be fulfilled not only by Li-ion devices, as in the case of DNN training, but also by CMOS floating gate memory [55], RRAM [137], and PCM [148] devices thanks to their ability to finely tune the conductance with analog precision to encode the stored weights.

On the other hand, hardware implementation of brain-inspired SNNs for sensors or embedded systems primarily requires high energy efficiency to enable sensory information processing for long times even in limited-energy environments. The high endurance of synaptic and neuron devices is also strongly required in that SNN operation relies on a learning approach based on continuous synaptic updates and continuous reset operations of integrate-and-fire neurons upon fire events. In addition to these features, a high resistance window could be useful for accurate continual learning although multilevel weight storage could be not strictly needed, as shown by significant applications using binary stochastic memory devices, such as STT-MRAM. Therefore, both NOR Flash memory [57], despite higher operating voltages, and all the memristive emerging devices show a strong potential for hardware implementation of SNNs emulating the efficiency and 3D architecture of the biological brain.

Although some limitations currently hinder the large-scale industrialization of memory-centric neuromorphic technology, the rich physics of memory devices can also offer additional biologically inspired functionalities and more. For instance, besides synaptic implementation, integrate-and-fire neuron functionality has been recently demonstrated in various types of memristive devices, including RRAM [180], volatile RRAM [175], Mott memristor [181], PCM [182], STT-MRAM [183,184], SOT-MRAM [126], and paramagnetic MTJs [185], thus opening the way for hardware implementation of high-density fully memristive neural networks with a high area and energy efficiency. Also, thanks to the short-term memory effects observed in some materials, a more realistic implementation of biological synaptic behavior taking into account the impact of spatiotemporal patterns has been achieved [171,172,173]. Moving from the standpoint of the device to that of the system, in-memory computing with memristive devices is opening the way to the exploration of new learning algorithms exhibiting strong similarity with human experience, such as reinforcement learning [186], which has already been shown to enable complex tasks [187].

Finally, memristive devices are receiving increasing interest for the development of other computing concepts by neuromorphic networks with high computational power, such as the Hopfield recurrent neural network [188]. Although high acceleration performance has been achieved for the solution of hard constraint-satisfaction problems (CSPs), such as the Sudoku puzzle, via CMOS-based circuits [189], FPGA [190], and quantum computing circuits [191], the use of memristive devices in crossbar-based neural networks can further speed up computation by the introduction of a key resource as the noise [192] without the requirement of additional sources [193]. Moreover, very recent studies have also evidenced the strong potential of memristive devices for the execution of complex algebraic tasks, including the solution of linear systems and differential equations, such as the Schrödinger and Fourier equations, in crossbar arrays in only one computational step [16], thus overcoming the latency of iterative approaches [15]. Therefore, these achievements suggest CMOS/memristive devices as enablers of novel high-efficiency computing paradigms capable of revolutionizing many fields of our society.

## 7. Conclusions

This work provides an overview of the most promising devices for neuromorphic computing covering both CMOS and memristive device concepts. Physical MVM in memristive/CMOS crossbar arrays implementing DNNs and SNNs has enabled both fundamental cognitive applications, such as image and speech recognition, and the solution of algebraic and constraint-satisfaction problems in hardware. These milestones can thus pave the way to highly powerful and energy-efficient neuromorphic hardware based on CMOS/memristive technologies, making AI increasingly pervasive in future society.

## Figures and Tables

**Table 1:** Comparison of key features exhibited by CMOS mainstream memory devices and memristive emerging memory devices under investigation to implement neuromorphic computing in hardware. Adapted from [35].

Technology	CMOS Mainstream Memories		Memristive Emerging Memories						
	NOR Flash	NAND Flash	RRAM	PCM	STT-MRAM	FeRAM	FeFET	SOT-MRAM	Li-ion
ON/OFF Ratio	10^4^	10^4^	10–10^2^	10^2^–10^4^	1.5-2	10^2^–10^3^	5–50	1.5–2	40–10^3^
Multilevel operation	2 bit	4 bit	2 bit	2 bit	1 bit	1 bit	5 bit	1 bit	10 bit
Write voltage	<10 V	>10 V	<3V	<3V	<1.5 V	<3 V	<5 V	<1.5 V	<1 V
Write time	1–10 μs	0.1–1 ms	<10 ns	~50 ns	<10 ns	~30 ns	~10 ns	<10 ns	<10 ns
Read time	~50 ns	~10 μs	<10 ns	<10 ns	<10 ns	<10 ns	~10 ns	<10 ns	<10 ns
Stand-by power	Low	Low	Low	Low	Low	Low	Low	Low	Low
Write energy (J/bit)	~100 pJ	~10 fJ	0.1–1 pJ	10 pJ	~100 fJ	~100 fJ	<1 fJ	<100 fJ	~100 fJ
Linearity	Low	Low	Low	Low	None	None	Low	None	High
Drift	No	No	Weak	Yes	No	No	No	No	No
Integration density	High	Very High	High	High	High	Low	High	High	Low
Retention	Long	Long	Medium	Long	Medium	Long	Long	Medium	-
Endurance	10^5^	10^4^	10^5^–10^8^	10^6^–10^9^	10^15^	10^10^	>10^5^	>10^15^	>10^5^
Suitability for DNN training	No	No	No	No	No	No	Moderate	No	Yes
Suitability for DNN inference	Yes	Yes	Moderate	Yes	No	No	Yes	No	Yes
Suitability for SNN applications	Yes	No	Yes	Yes	Moderate	Yes	Yes	Moderate	Moderate
